# Impacts of climate change on water resources in the major countries along the Belt and Road

**DOI:** 10.7717/peerj.12201

**Published:** 2021-10-12

**Authors:** Panpan Du, Ming Xu, Renqiang Li

**Affiliations:** 1 Key Laboratory of Ecosystem Network Observation and Modeling, Institute of Geographic Sciences and Natural Resources Research, Chinese Academy of Sciences, Beijing, China; 2 University of Chinese Academy of Sciences, Beijing, China; 3 Sino-Danish Centre for Education and Research (SDC), Beijing, China

**Keywords:** Climate change, Water shortage, Global Climate Model (GCM), Runoff, Downscaling

## Abstract

**Background:**

Climate change has altered global hydrological cycles mainly due to changes in temperature and precipitation, which may exacerbate the global and regional water shortage issues, especially in the countries along the Belt and Road (B&R).

**Methods:**

In this paper, we assessed water supply, demand, and stress under three climate change scenarios in the major countries along the Belt and Road. We ensembled ten Global Climate Model (GCM) runoff data and downscaled it to a finer resolution of 0.1° × 0.1° by the random forest model.

**Results:**

Our results showed that the GCM runoff was highly correlated with the FAO renewable water resources and thus could be used to estimate water supply. Climate change would increase water supply by 4.85%, 5.18%, 8.16% and water demand by 1.45%, 1.68%, 2.36% under RCP 2.6, 4.5, and 8.5 scenarios by 2050s, respectively. As a result, climate change will, in general, have little impact on water stress in the B&R countries as a whole. However, climate change will make future water resources more unevenly distributed among the B&R countries and regions, exacerbating water stress in some countries, especially in Central Asia and West Asia. Our results are informative for water resource managers and policymakers in the B&R countries to make sustainable water management strategies under future climate change.

## Introduction

Water is one of the most vital natural resources for almost all societal and economic activities. The global water resources per capita have declined dramatically with the rapid growth of population and economy. The global freshwater resources per capita have decreased from 13,360.32 m^3^ in 1962 to 5,925.67 m^3^ in 2014 (World Bank, 2016). Global climate change may also exacerbate water shortage due to the decrease in precipitation in many regions of the world (Hanasaki et al., 2013b; Cramer et al., 2014). Meanwhile, global warming may also further increase water demands mainly due to the increase in evaporation and transpiration by plants (Praskievicz & Chang, 2009; Alkama, Kageyama & Ramstein, 2010; Chang, Praskievicz & Parandvash, 2014). For instance, a recent study showed that 1 °C increase in air temperature would increase residential water demand by 3.78 L per capita per day (Al-Zubari et al., 2018). Therefore, it is of great significance to explore the impacts of climate change on water resources in the future. Previous studies assessed global water stress by two measures: “Water Crowding Index” (WCI, annual water resources per capita) and “Water Stress Index” (WSI, the ratio of water demands to resources) showing that approximate 35–60% of global population were under moderate and severe water stress in 1990s at different spatial scale (Vörösmarty et al., 2000; Arnell & Lloyd-Hughes, 2014; Schewe et al., 2014). A number of studies have investigated the role of climate change on future water resources showing that future water stress increase was driven mainly by socio-economic factors and the impacts of climate change regionally varied (Alcamo, Martina & Michael, 2007; Gerten et al., 2011; Hanasaki et al., 2013b; Gosling & Arnell, 2016). However, most studies ignored climate change impacts on future water demands.

The Belt and Road (B&R), also known as “the Silk Road Economic Belt and the 21st-Century Maritime Silk Road”, is a global initiative led by China (Lin, 2015; Zhang et al., 2018). As of today, more than 130 countries have joined the Belt and Road initiative and the number is still growing. One hundred and fifteen of the B&R countries are developing countries and 26 of them have the annual available water resources less than 1,000 cubic meters per capita, a threshold of severe water shortage used by the FAO (FAO, 2016). There have been a limited number of studies on water resources in the B&R countries. Zuo, Hao & Liu (2018) estimated the amount of water resources along the B&R and found that Southeast Asia accounted for 46.88% of the total water resources in the B&R countries, while East and North Africa merely accounted for less than 1%. Yang et al. (2019) investigated the current water resources status and water use potential of the B&R countries by FAO AQUASTAT data and found that the exploitation potential of water resources was low in the Arabian Peninsula, and high in Central and Eastern Europe and Southeast Asia. Water resources in some regions, such as Central Asia (Hao et al., 2018), Africa (Li, Liu & Hao, 2018), South Asia (Wei et al., 2018), Europe (Han & Ma, 2018), Southeast Asia (Li et al., 2018), of the Belt and Road were also examined in previous studies.

However, there have been no studies reported in the literature to examine future water resources across the Belt and Road under the impacts of climate change. In this study, we assessed the supply and demand of water resources for 122 countries along the Belt and Road under different climate change scenarios for the mid-21st century. Our results will provide foundational information to policymakers to make adequate and sustainable water management strategies for the B&R in the face of climate change.

## Materials & Methods

### Water resources estimation and downscaling of GCM runoff data

Water resources can be quantified in multiple ways, such as stream flow, surface runoff, or total runoff (Weiland et al., 2011; Alkama et al., 2013; Schewe et al., 2014). In the current study, we used total runoff to represent the overall renewable water resources because climate change will affect many hydrological processes including ground water which has been extracted in many countries, especially for irrigation use. The runoff is also known as “Blue Water” and “Available Freshwater” (Wada et al., 2013; Arnell & Lloyd-Hughes, 2014).

In this study, the total runoff data was obtained from the Coupled Model Intercomparison Project (CMIP5) of the IPCC fifth assessment (Taylor, Stouffer & Meehl, 2012; Zhang et al., 2014; IPCC Working group 1, 2014). Those runoff data were also used in previous studies to improve water resources management in Brazil (Gondim et al., 2018). The CMIP5 dataset has advantages for the current study because: (1) many of the GCM models had incorporated detailed land surface processes including hydrological cycles; (2) the models had consistent outputs for the current (near present) and future climate conditions based on the well-studied scenarios of future socioeconomic and climatic changes; and (3) the dataset had simulation outputs from dozens of models which made it possible to ensemble multi-model outputs to increase our confidence in the future projections (Zhou et al., 2014). Moreover, simulated runoff from different GCM models has been validated at global, continental and basin scales in previous studies with observations (Decharme & Douville, 2007; Alkama et al., 2010; Decharme et al., 2010; Hagemann, Loew & Andersson, 2013; Voldoire et al., 2013; Gao & Zhao, 2020). To predict the future climate, the climate modeling groups had designed four Representative Concentration Pathways (RCPs), which presented different trajectories of anthropogenic climate change in terms of radiative forcing (Vuuren et al., 2011; IPCC Working group 1, 2014). These four RCP scenarios included a low emission scenario (RCP 2.6), two medium stabilization scenarios (RCP 4.5 and RCP 6.0) and one high emission scenario (RCP 8.5). We selected three out of the four scenarios (RCP 2.6, 4.5 and 8.5) to examine the impacts of climate change on water resources which have been widely used in previous studies (Kharin et al., 2013; Su et al., 2016; Wang et al., 2020).

However, the spatial resolution of most simulation results from CMIP5 GCM was too coarse to be used directly in climate change impacts studies. Downscaling techniques offer an alternative to develop finer resolution outputs (Akinsanola et al., 2018). Of many downscaling techniques statistical downscaling has been widely used in climate change studies due to its simplicity and ease of implementation (Hessami et al., 2008; Monjo et al., 2016; Su et al., 2016). Besides, the random forest model has been successfully used to downscale GCM data in previous studies due to the ability to handle large datasets with correlated conditional variables and the inbuilt variable importance evaluation (Pang et al., 2017). Therefore, we used the random forest (RF) model to downscale the GCM outputs of runoff to the spatial resolution of 0.1 × 0.1 degree.

In the current study, we used the runoff data of ten GCMs in the CMIP5 project (Table 1). We first downloaded the daily runoff data for the current (1986–2005) and the future (2046–2065) from the CMIP5 website (Table S1). Then we ensembled the data by taking the mean using the R software which has been commonly used in previous climate change studies (Milly, Dunne & Vecchia, 2005; Shen et al., 2008; Su et al., 2016). We further averaged the ensembled daily data along the temporal scale for the two periods (1986–2005 and 2046–2065) respectively. Then we downscaled the coarse runoff data to a fine resolution (0.1° × 0.1°) with three steps. In the first step, we used the RF model to downscale the current runoff data by a high resolution (0.1° × 0.1°) climate dataset, the WorldClim dataset (Fick & Hijmans, 2017). The driving variables in constructing the RF model included the average, maximum and minimum temperature, precipitation, solar radiation, wind, water vapor and normalized difference vegetation index (NDVI). To consider the effects of seasonal variability of climate on runoff, we used the multi-year average and the January and July data of each variable to drive the downscaling model. The WorldClim data was downloaded from the WorldClim website (Table S1). The NDVI data was downloaded from the MODIS datasets and processed in GIS to get the multi-year (2000–2005) average. The Nash-Sutcliffe (Nash) coefficient was used to evaluate the performance of the model. In the second step, we calculated the difference between the current and future runoff using the coarse GCMs data, also known as the delta method (Hay, Wilby & Leavesley, 2000). In the third step, we added the difference (delta) of runoff to the downscaled current runoff of each pixel to get the future (2046–2065, representing 2,055) high resolution runoff data.

**Table 1:** The basic information of selected GCMs.

Model name	Institute	Resolution (°)
CanESM2	Canadian Centre for Climate Modelling and Analysis	2.81 × 2.78
CNRM_CM5	National Centre for Meteorological Research	1.41 × 1.41
CSIRO-Mk3.6.0	Commonwealth Scientific and Industrial Research Organization	1.88 × 1.86
MIR-CGCM3	Meteorological Research Institute	1.13 × 1.12
MIROC-ESM	Atmosphere and Ocean Research Institute	2.81 × 2.79
MIROC-ESM-CHEN	National Institute for Environmental Studies	2.81 × 2.79
MPI-ESM-MR	Max Planck Institute for Meteorology	1.88 × 1.86
GFDL-ESM2M	Geophysical Fluid Dynamics Laboratory	2.48 × 1.98
GFDL-ESM2G	Geophysical Fluid Dynamics Laboratory	2.79 × 3.00
FGOALS-g2	Institute of Atmospheric Physics, Chinese Academy of Sciences	2.79 × 3.00

Previous studies have reported that due to lack of complete information about atmospheric phenomena, some assumptions were made during the development of GCMs, which caused systematic bias in GCM data (Bring et al., 2015; Noor et al., 2019). To correct these biases, we used the country-level renewable water resources compiled by the FAO (Hoogeveen et al., 2015; FAO, 2016). We compared two methods for this purpose. The first one is the change factors (CF) which is the ratio of FAO runoff to the modeled runoff (Taye et al., 2018). The second one is a regression method which is a linear relationship between the FAO runoff and the modeled runoff of 176 countries excluding some small island countries.

### Projection of water demands under future climate change

Country-based water demands were divided into three categories: agricultural, residential and industrial water demand as classified by the FAO. The current country-level water demands of these sectors were obtained from the FAO AQUASTAT, a global database of water resources developed by the FAO mainly based on countrywide statistics (FAO, 2016). We interpolated the missing data by a linear interpolation method since AQUASTAT provided country-based data at a 5-year interval. A full detail of the interpolation method can be found in Liu et al. (2016).

For agricultural water demand, we did not differentiate the livestock water demand with the irrigation water because irrigation water constitutes more than 90% of the total agricultural water demand (Huang et al., 2018). The future agricultural water demand is driven by the changes of cropland area and irrigation rate per unit of cropland area. We ignored the change of irrigation rate per unit cropland area caused by climate change due to insufficient knowledge on this complex issue. Many studies have shown that increasing atmospheric CO_2_ concentration will increase plant water use efficiency and thus reduce agricultural water demand (Alkama, Kageyama & Ramstein, 2010; Rajagopalan et al., 2018; Jans et al., 2021). Whereas, studies also have shown that increasing temperature will increase cropland water use due to the increase of evapotranspiration (Mahmood et al., 2006; Wada et al., 2013; Sylla et al., 2018). The net effects of these two competing processes are not conclusive. However, most studies have shown that these two processes are on the same level, indicating that the net effect on water demand is minor (Lee & Huang, 2014; Jans et al., 2021).

The residential water demand is affected by many factors, such as economic growth, technological evolution and global warming, which is indicated by residential water intensity (given as residential water demand per day per capita) (Shen et al., 2008; Parkinson et al., 2016). We calculated the baseline residential water demand (RWD, m^3^·yr^−1^) as in Hanasaki et al. (2013a):

(1) $${\rm RWD} = {\rm POP} \times \left( {{{\rm i}_{{\rm resi},{\rm t}0}} + {{\rm s}_{{\rm resi}}} \times \left( {{\rm t} - {{\rm t}_0}} \right)} \right) \times 0.365$$

where POP is the population, ${{\rm i}_{{\rm resi},{\rm t}0}}$ is the residential water intensity for the base year (L·day^−1^·capita^−1^), ${{\rm s}_{{\rm resi}}}$ is the growth rate of ${{\rm i}_{{\rm resi},{\rm t}0}}$ (L·day^−1^·capita^−1^·yr^−1^), and ${\rm t}$ is the number of year in the future.

The gridded population and GDP data in the 2050s were obtained from Murakami & Yamagata (2019). In this method, the growth rate of residential water intensity (L·day^−1^·capita^−1^·yr^−1^) of each country was computed by dividing the countries into high-income (per capita GDP > 2,000 USD) and low-income (per capita GDP < 2,000 USD) categories. In addition, global warming will also stimulate residential water demand. We calculated the global warming effect on residential water demand by assuming future residential water demand will increase by 1% as daily maximum air temperature increases 1 °C according to the previous studies (Wang & Yang, 2010; Polebitski & Palmer, 2010; Al-Zubari et al., 2018). The daily maximum temperature data was also obtained from the WorldClim database.

As with agricultural and residential water demands, future industrial water demand is also affected by socioeconomic conditions and climate change. Similarly, we used air temperature to calculate the global warming effect on the industrial water demand. We assumed that the industrial water demand would increase by 2% with temperature increase of 1 °C according to previous studies (Wang & Yang, 2010; Khan et al., 2012).

### Water stress index

We used the water stress to assess the impacts of climate change on water shortage. The water stress index has been widely used (Vörösmarty et al., 2000; Oki et al., 2001; Gosling & Arnell, 2016) and it was computed as:

(2) $${\rm WSI} = \displaystyle{{{\rm TWD}} \over {{\rm RWR\; }}}$$

(3) $${\rm TWD} = {\rm AWD} + {\rm RWD} + {\rm IWD}$$

where TWD is the total water demands (km^3^·yr^−1^), consisting of agricultural water demand (AWD), residential water demand (RWD) and industrial water demand (IWD) and RWR is renewable water resources (km^3^·yr^−1^). The classification standard of the WSI is as follows: WSI < 0.20: no water stress; 0.20 < WSI ≤ 0.60: low water stress; 0.60 < WSI ≤ 0.75: moderate water stress and WSI > 0.75: high water stress.

## Results

### Downscaling and bias correction of the GCM runoff data

**Figure 1:**
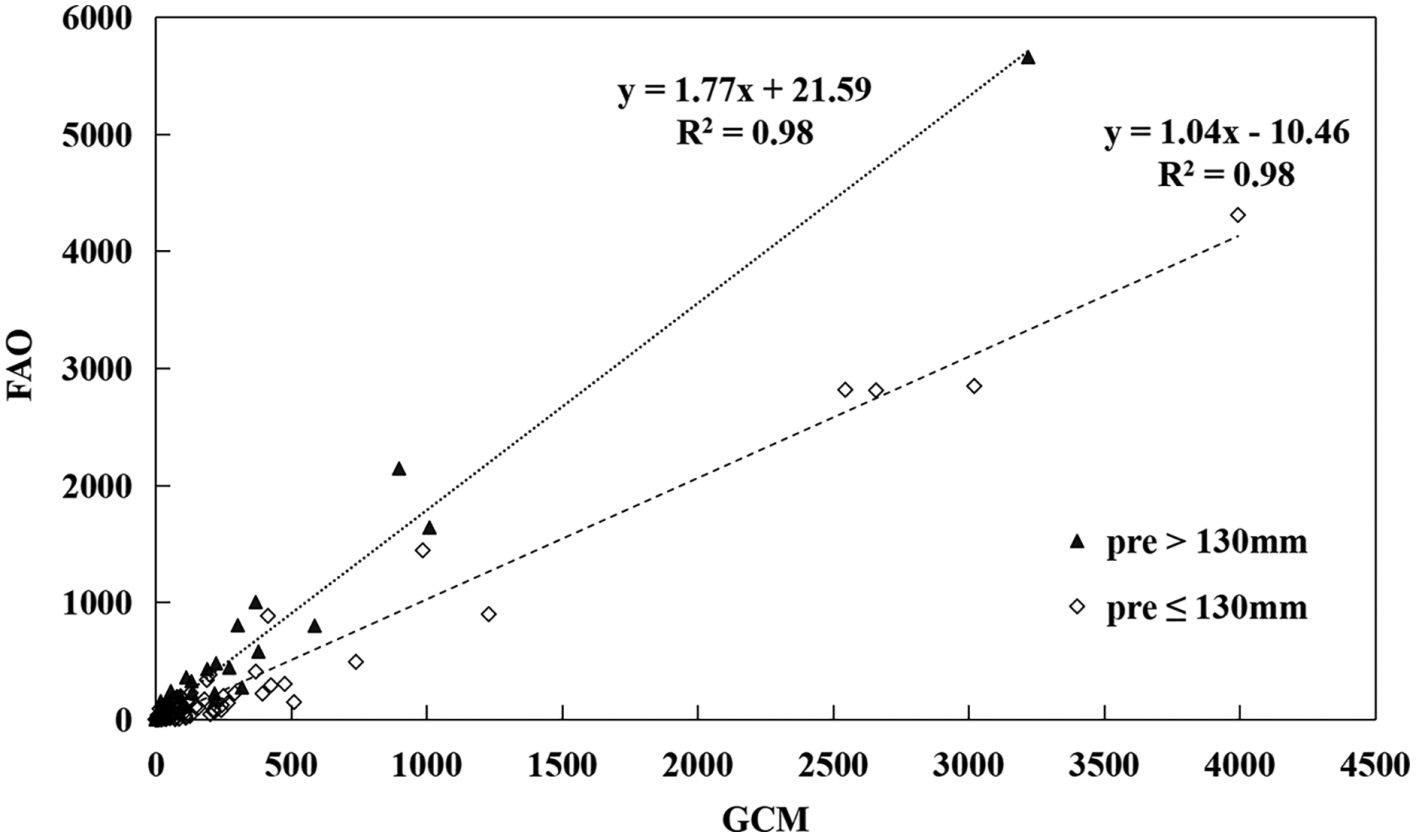
Regression analyses of the country-based water resources between the FAO and GCMs estimates.

We found that the RF model was quite robust in downscaling the GCM runoff data. The average of Nash coefficient reached 0.98 for training datasets and 0.94 for independent validation datasets. We found that the modeled global runoff average between 1986 and 2005 was 35,692.05 km^3^·yr^−1^, which was 16.64% lower than the FAO global water resources of 42,820.22 km^3^·yr^−1^ for the same period (FAO, 2016) due to systematic bias in GCM data and uncertainties from the random forest model. However, we also found that the modeled runoff was highly correlated with the FAO country-based water resources (Fig. 1). To make the modeled runoff comparable with the FAO renewable water resource data, we used the ratio (FAO renewable water resources divided by the modeled runoff for each country) as a correction factor to keep the current and future runoff data in line with the FAO statistical water resources data. We also developed regression models to correct the bias of the modeled runoff data (Fig. 1). Although the regression models had very high coefficients of determination (R^2^), we found that the regression models overestimated water resources in the dry areas and underestimated in the wet regions (Fig. 1). Since many water shortage countries in the B&R are located in the dry areas with a small amount of runoff, the regression model correction would make a relatively larger bias to those countries where climate change may have severe impacts on water resources, the focal areas of the current study. Therefore, we used the simple ratio method instead of the regression models to calculate the current and future renewable water resources for each country based on the modeled total runoff.

### Current status of water resources in major countries along the Belt and Road

We found that the renewable water resources in the B&R countries were significantly lower than that of the global average. Our results showed that the “current” (1986–2005) renewable water resources in the B&R countries were 22997.05 km^3^·yr^−1^ accounting for 53.86% of the global total water resources, though the total land area of the B&R countries accounted for 66.62% of the global land area and 63.49% of the global population. The average water resources per unit area in the B&R countries were 210.16 mm·yr^−1^ which were 19.38% lower than the global average of 261.95 mm·yr^−1^. The renewable water resources per capita in the B&R countries were 5977.14 m^3^·yr^−1^ which were 15.16% lower than the global average of 7,045.55 m^3^·yr^−1^.

**Figure 2:**
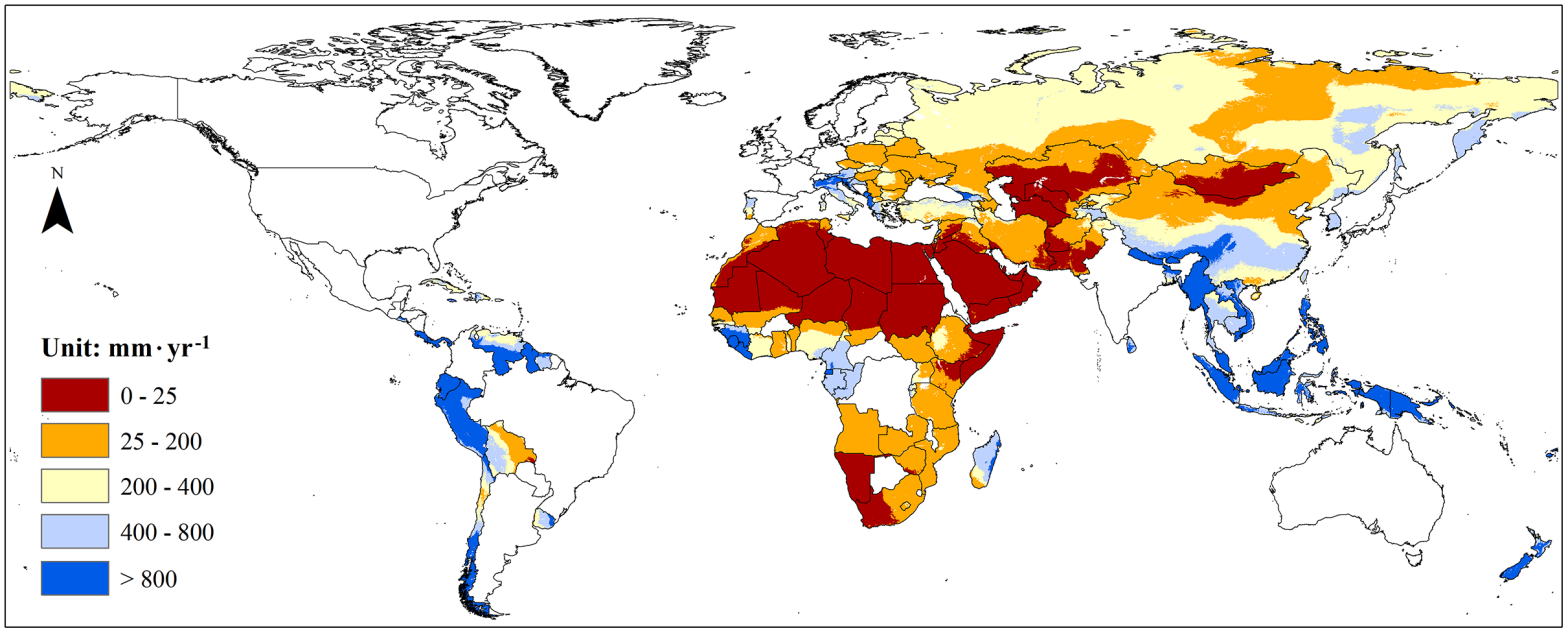
The average renewable water resources (1986–2005) of the major countries in the Belt and Road.

Moreover, we found that water resources were unevenly distributed among the B&R countries (Fig. 2). Southeast Asia had the richest water resources with an average of 1,108.87 mm·yr^−1^, while the poorest water resources region was North Africa with an average of 2.06 mm·yr^−1^. The areal based water resources in Southeast Asia were about 538 times that of North Africa. The highest water resources per capita among the B&R countries were found in South and Central America with an average of 39,901 m^3^·yr^−1^ compared with the lowest of 340 m^3^·yr^−1^ found in North Africa.

### Climate change impacts on the supply of water resources

We found that climate change would slightly increase total renewable water resources amount of the Belt and Road by the mid-21st century. The renewable water resources of all the B&R countries may increase from the current 22,997.07 km^3^·yr^−1^ to 24,113.05 km^3^·yr^−1^, an increase of 4.85%, under RCP 2.6, a low climate change scenario. Under RCP4.5, a moderate climate change scenario, water resources in the B&R countries may increase by 5.18%, reaching 24,190.86 km^3^·yr^−1^. The renewable water resources in the B&R countries may see a slightly greater increase of 8.16%, with an amount of 24,874.33 km^3^·yr^−1^, under RCP8.5, the most aggressive climate change scenario in the IPCC fifth assessment.

**Figure 3:**
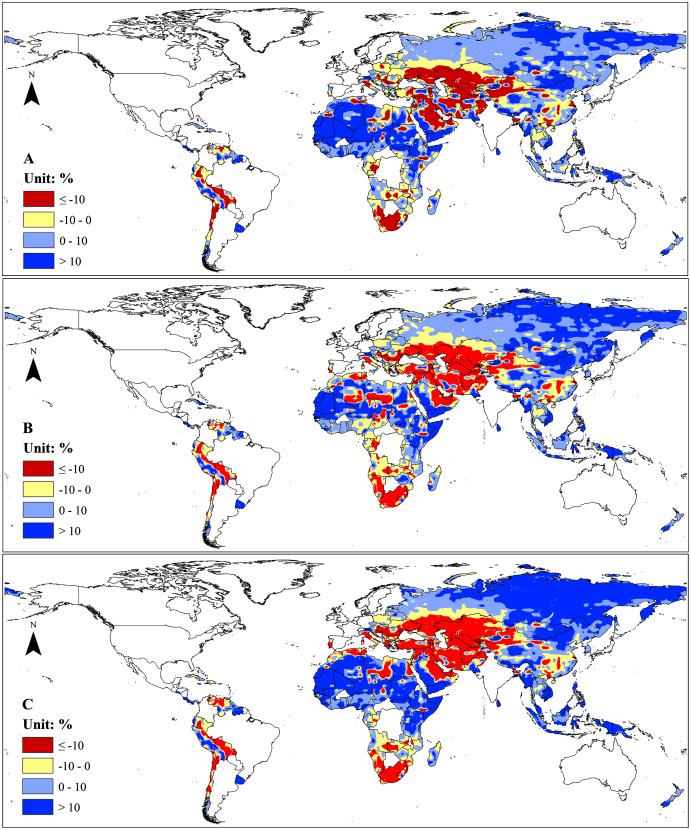
Change of renewable water resources under different climate change scenarios by 2050 compared with the baseline (1986–2005). (A) RCP 2.6. (B) RCP 4.5. (C) RCP 8.5.

However, we found that climate change would exacerbate the uneven distribution of water resources among the B&R countries with exceptions in North Africa and the Mongolia Plateau (Fig. 3). Future climate change may decrease water resources in Central and West Asia, South Africa, South China, Chile, Bolivia, and Venezuela by more than 10% (Fig. 3). Meanwhile, climate change may also increase water resources in North Africa, Russia, Mongolia Plateau, West China, Southeast Asia, Peru and Uruguay (Table S2). Future climate change may increase water resources in the current water-rich regions and decrease in the water shortage regions. The proportion of water shortage areas with water resources lower than 25 mm·yr^−1^ will slightly increase from 27.71% up to 28.19% and the proportion of areas with water resources greater than 800 mm·yr^−1^ will increase from the current 9.98% up to 10.72%. We found that different climate change scenarios had similar effects on water resources in terms of amount and spatial distribution though the impacts of RCP 8.5 was slightly more severe than those of RCP 2.6 and RCP 4.5.

### Climate change impacts on water demands

In addition to water supply, climate change may also increase water demands in various sectors. Our results showed that the current total water demands in the B&R countries were 2,010.05 km^3^·yr^−1^, which were about 56% of the global water demands (3,595.37 km^3^·yr^−1^). Agricultural, residential and industrial water demand in the B&R countries accounted for 77.58%, 9.05% and 13.37%, respectively, of the total water demands of those countries. We found that future climate change would have a minor impact on water demands in the B&R with an increase of 1.45%, 1.68%, and 2.36% respectively under RCP 2.6, RCP 4.5, and RCP 8.5. However, the industrial and residential water demands would increase much faster than the total water demands due to the large share of agricultural water demand which was assumed unchanged with climate change in the current study. The industrial water demand will increase by 7.91%, 9.15%, and 10.58% respectively under RCP 2.6, RCP 4.5, and RCP 8.5. The residential water demand will increase by 4.33%, 5.04%, and 10.47% under RCP 2.6, RCP 4.5, and RCP 8.5, respectively.

**Figure 4:**
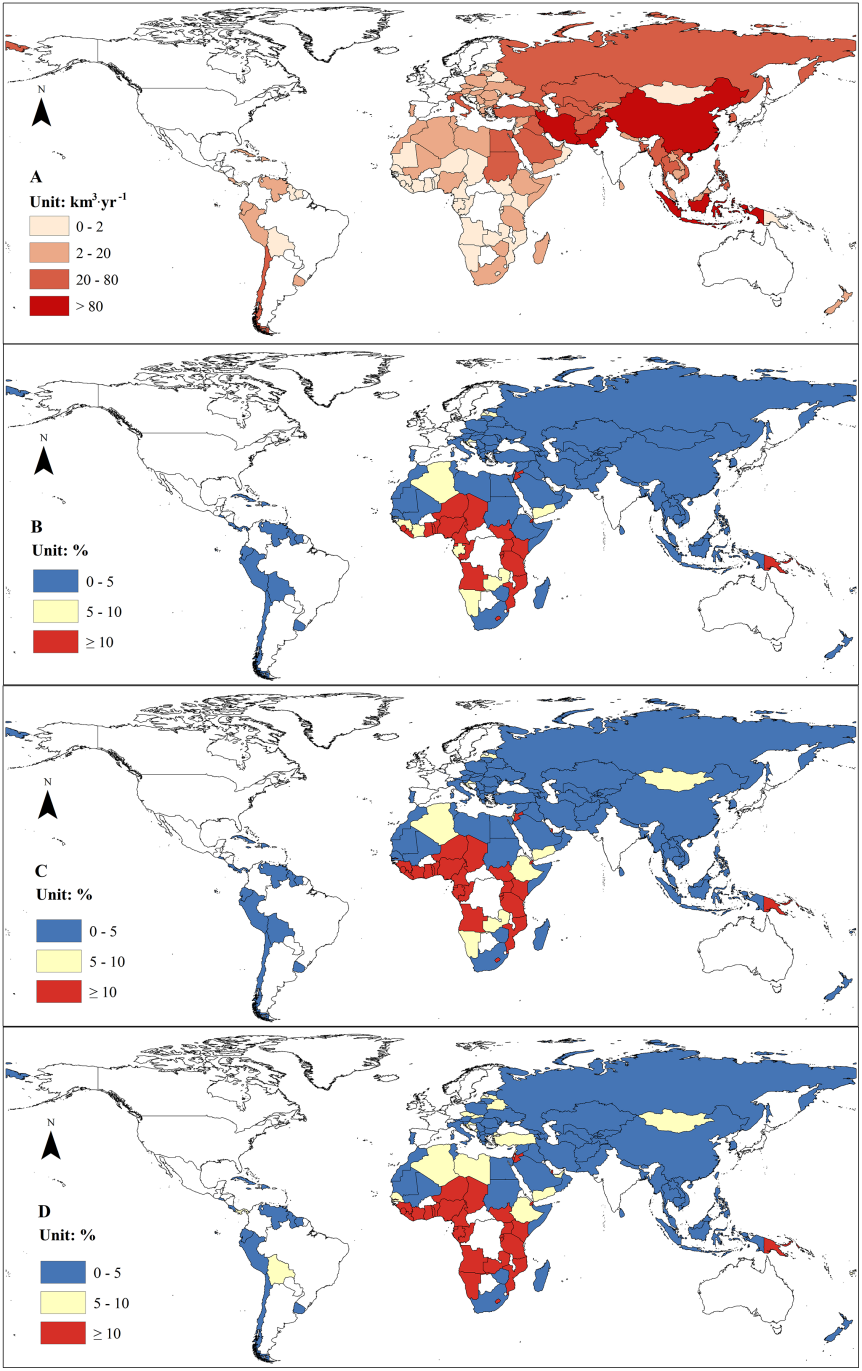
Country-based water demand (1986–2005) and change of the water demand under different climate change scenarios. (A) Country-based annual water demand (1986–2005). (B) RCP 2.6. (C) RCP 4.5. (D) RCP 8.5.

Spatially, the high increase of water demands driven by future climate change was found in Central Africa and Papua New Guinean where the climate change induced more than 10% increase in water demands, while the growth rates of water demands induced by climate change may be less than 5% in most of the B&R countries in Asia, Europe, South America, and North and South Africa (Fig. 4). Mongolia and a few countries in Africa may experience 5–10% increase of water demands under different climate change scenarios (Fig. 4).

### Climate change impacts on water stress in the B&R countries

**Figure 5:**
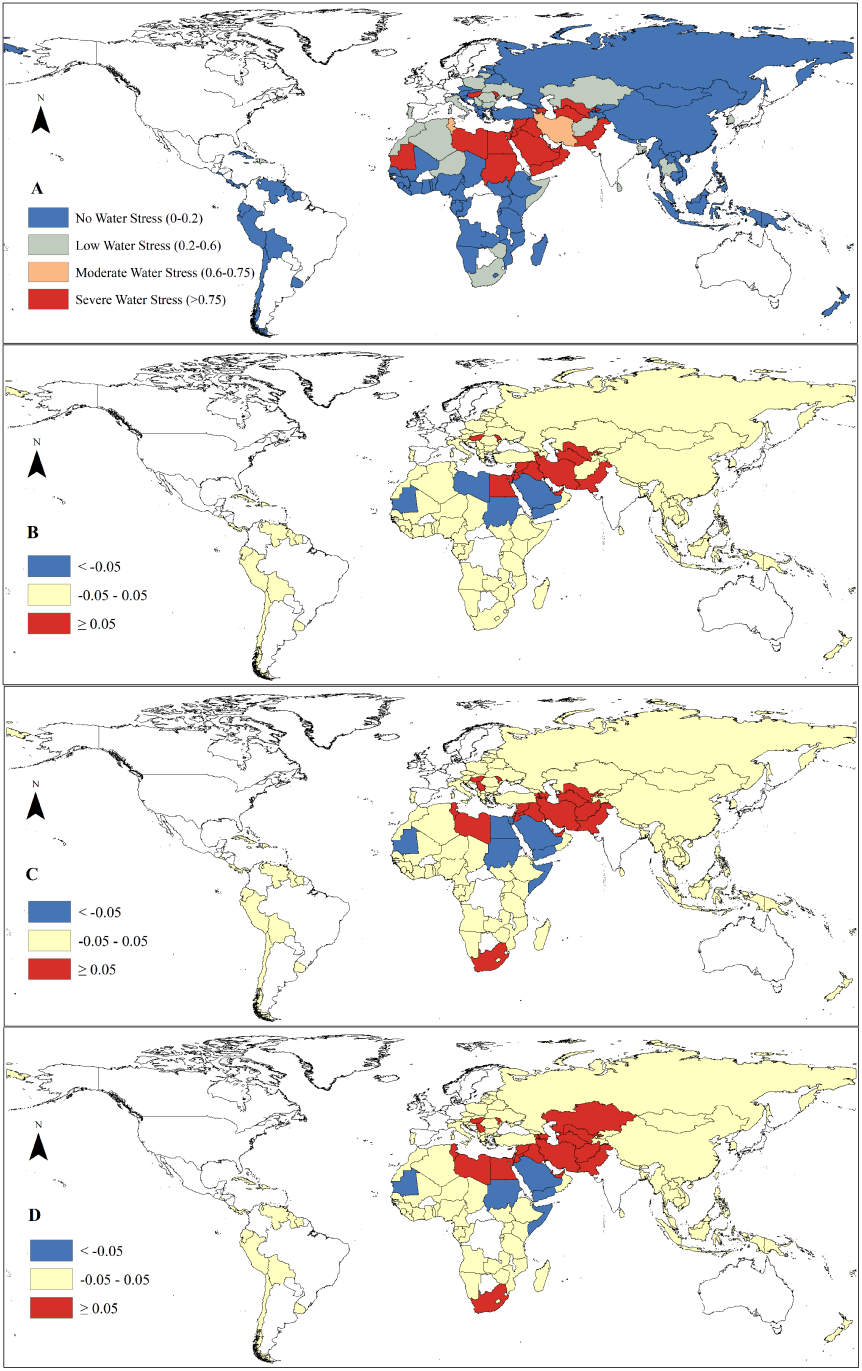
Country-based water stress index (1986-2005) and changes of water stress index under different climate change scenarios. (A) Country-based water stress index (1986–2005). (B) RCP 2.6. (C) RCP 4.5. (D) RCP 8.5.

We found that climate change alone would have little impact on the overall status of water resources in the B&R countries with a slight increase in the total renewable water resources. Climate change will increase water supply by 4.85–8.16% and water demand by 1.45–2.36% without considering the climate change impacts on agricultural water demand. However, climate change will enhance the uneven distribution of water resources among the B&R countries and regions, exacerbating water stress in some countries, such as Iran, Tunisia, Afghanistan, Tajikistan, and Turkey, most of which have already suffered from water shortage (Fig. 5). However, most of the B&R countries will experience little change in water stress under future climate change. We found that water stress will be mitigated in some countries, such as Saudi Arabia, Yemen, Sudan, and Mauritania, under climate change. The change of water stress in some other countries depends on climate change scenarios under combined impacts on both the water supply and water demand. Libya may experience a reduction in water stress under RCP 2.6 owing to the substantial increase of water supply (Table S2), but an increase in water stress under RCP 4.5 and RCP 8.5 (Fig. 5). South Africa may see little change in water stress under RCP 2.6 but an exacerbated water stress under RCP 4.5 and RCP 8.5 with high decrease rate of water supply and increase rate of water demand (Table S2, Fig. 4). Egypt, however, may experience an increase in water stress under RCP 2.6 and RCP 8.5, but a decrease in water stress under RCP 4.5 (Fig. 5) due to higher increase rate of water supply than demand (Table S2, Fig. 4).

## Discussion

### GCM runoff data

**Figure 6:**
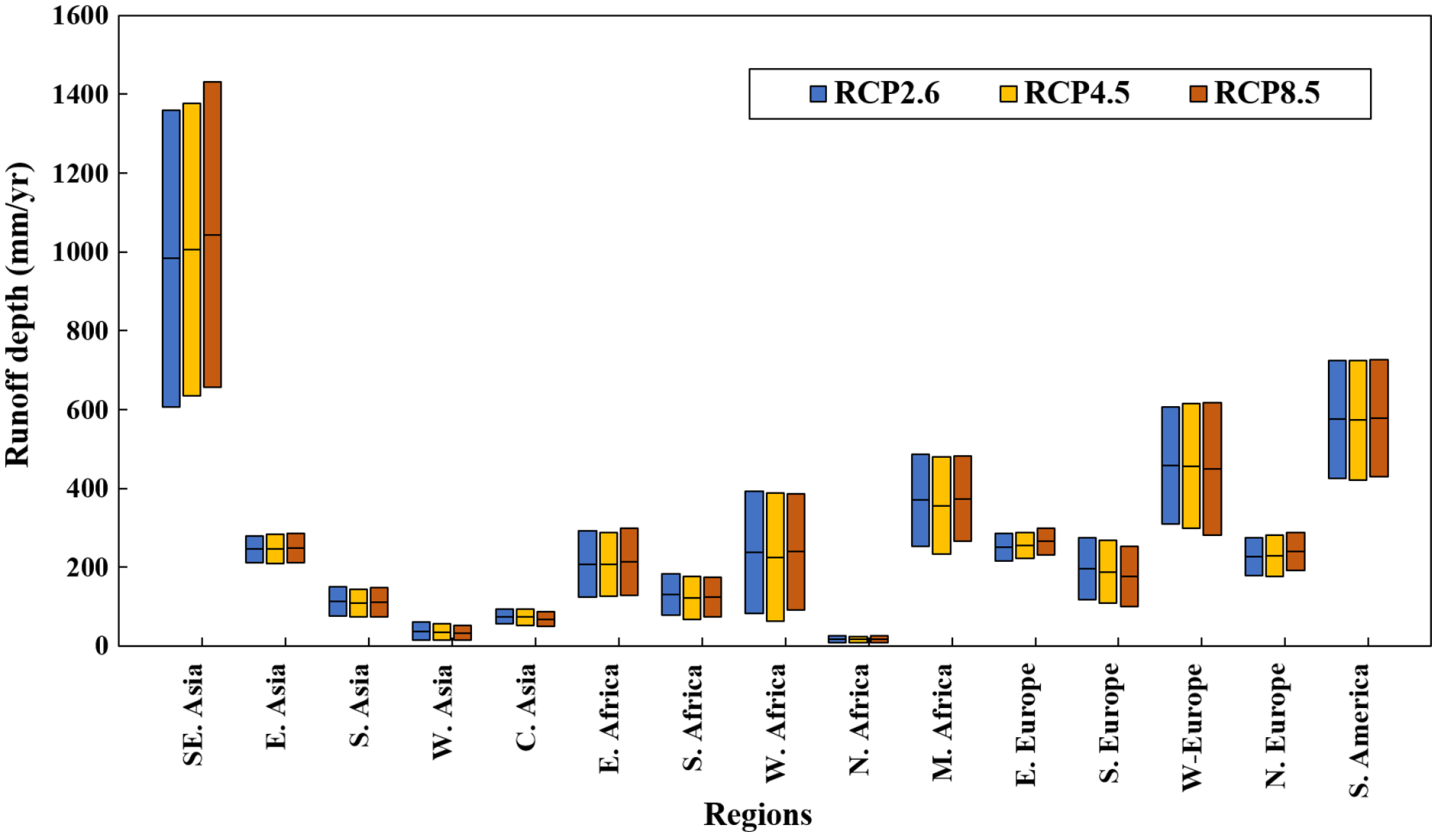
Average runoff depth and standard deviation among ten GCMs of different regions in the Belt and Road under RCP 2.6, RCP 4.5 and RCP 8.5. The horizontal black lines the average runoff depth, and the boxes give the range of one standard deviation. The Belt and Road countries were divided into 15 regions. New Zealand and seven small island countries in Central America were not included.

In this paper, we developed a high-resolution global runoff dataset using the CMIP5 GCM outputs by downscaling the GCM runoff data with the random forest model. We found that the GCM runoff data was highly correlated with the FAO statistics of the renewable water resources. Previous studies compared the CMIP5 GCM runoff with observed runoff in 161 river basins across the globe and found that the GCM runoff data was generally comparable with the observations (Alkama et al., 2013). However, other studies found that the GCM runoff data was significantly different from the observed runoff in six river basins in Europe and North America (Bring et al., 2015). The results of the latter study might be biased because the river basins used in this study had a small area compared with the coarse resolution of the GCM outputs. In this current study, we downscaled the GCM runoff to a finer resolution to avoid the bias and mismatching between the modeled and observed runoffs. Our results showed that there was a high correlation between the downscaled GCM runoff and the FAO country-based observations of water resources (Fig. 1). The projection of runoff change was consistent with previous studies that runoff increases in high northern latitudes, Eastern Africa, and to decrease in the Mediterranean (Alkama et al., 2013; Schewe et al., 2014; Gosling & Arnell, 2016). Climate model projections, although rather consistent in terms of global scale, diverged at regional scale (Schewe et al., 2014). Therefore, we divided the Belt and Road countries into 15 regions and calculated the mean and standard deviation of ten GCMs (Fig. 6). We found that there are consistencies in arid regions such as Western Asia, Central Asia, and North Africa, the focal areas of the current study. High uncertainty was characteristic for the Southeast Asia, however, even under the projection of MIROC-ESM-CHEN of RCP 2.6 with the lowest increase trend, the water stress index of countries in Southeast Asia didn’t increase significantly, except for Thailand. The uncertainty from different GCMs didn’t influence the main results of this study.

### Climate change impacts on water stress in the B&R countries

In the current study, we examined the impacts of climate change on the supply and demand of the renewable water resources in the B&R countries and found that future climate change might have a minor impact on water resources. This is because climate change will increase water supply more than water demands in most of the countries (Figs. 3, 5). The increase of water supply is mainly attributed to the increase of precipitation which, on average, will increase by 4.62%, 5.59%, and 6.51% respectively under RCP 2.6, RCP 4.5, and RCP 8.5 by 2050s over the B&R countries. Meanwhile, evapotranspiration (ET) will increase 4.5%, 5.7%, and 5.7% respectively under those climate change scenarios. The smaller increase in ET is partially attributed to the increase in atmospheric CO_2_ concentration. Many studies have shown that elevated CO_2_ concentration will increase plant and ecosystem water use efficiency (Yano, Aydin & Haraguchi, 2007; Rajagopalan et al., 2018; Sylla et al., 2018). Our result showed that climate change would increase total runoff in the B&R countries, in line with a previous study which concluded that climate change will significantly boost streamflow by 2040 (Alkama et al., 2013).

In this study, we found that climate change may have little impact on the total demands of water resources in B&R countries. This is because agricultural water demand accounts for 77.58% of the total water demands in the B&R countries and we assumed that climate change had no impact on agricultural water demand. However, our results showed that climate change would considerably increase industrial and residential water demand (Table 2). The assumption that agricultural water demand does not change with climate change is rough but reasonable according to our current understanding of the complex plant-water interactions. Crop water demand under future climate change is mainly determined by two competing processes: rising temperature stimulating ET and elevated CO_2_ concentration improving water use efficiency.

**Table 2:** Water demand for three sectors during the period of 1986–2005 and under different climate change scenarios in 2050s.

Water resources (km^3^·yr^−1^)	Present	RCP 2.6	RCP 4.5	RCP 8.5
Agricultural demand	1,559.41	1,559.41	1559.41	1559.41
Residential demand	181.83	189.71	190.99	200.86
Industrial demand	268.81	290.07	293.40	297.40
Total water demands	2,010.05	2,039.19 (+1.45%)	2,043.80 (+1.68%)	2,057.67 (+2.36%)

So far, experimental and model simulation studies have shown that warming alone may increase water demands by 3.6–15% through the boost of ET (Wang et al., 2016; Sylla et al., 2018; Jans et al., 2021) and meanwhile, the CO_2_ effect alone will decrease water consumption by 4.1–12.2% through CO_2_-induced decrease of stomatal conductance (Yano, Aydin & Haraguchi, 2007; Xu et al., 2016; Nechifor & Winning, 2019). These two processes are roughly cancelled out. Indeed, a recent study found that climate change barely increased crop water demand considering both the temperature and CO_2_ effects (Jans et al., 2021). We acknowledge that the temperature-CO_2_ interactions on plant growth and water use are complex and may vary in different climates and cropping systems. Further studies are needed to refine the estimates of agricultural water demand under future climate change.

Although climate change may have little impact on water stress for the B&R countries as a whole, some of the countries may still suffer more water stress due to climate change, especially countries in Central and West Asia, which are currently facing water shortage problems (Fig. 5). Our results also showed that some of the B&R countries may even get “benefits” from future climate change (Fig. 5). In our analysis, we did not include the potential water demands driven by socioeconomic factors, such as population and economic growth, therefore, cares should be taken while interpreting the results for decision making on water resource adaptation to climate change. According to the most recent projections, the Shared Socioeconomic Pathways (SSPs), we found that water demands in the B&R countries would increase 22.22%, 25.59%, and 29.8% respectively under SSP1, SSP2, and SSP3 by the middle of this century. Apparently, socioeconomic factors had large impact on future water demands. However, climate change will have a considerable effect on water demands in some countries, especially the countries with rapid industrialization because climate change may significantly boost industrial water demands according to our analyses (Table 2). It is noted that many countries in the B&R including those currently having no water stress may suffer much more severe water stress in the future if climate change and socioeconomic factors are all considered together. To our knowledge, this study is the first to assess the impacts of climate change on water resources across all the B&R countries.

## Conclusions

In this paper, we developed a global runoff dataset by ensembling ten GCM outputs of total runoff and downscaled to a much finer resolution for analyzing climate change impacts on water resources and water stress in the B&R countries. We found that the GCM runoff was highly correlated with the FAO statistics of renewable water resources and thus could be reasonably used for further analyses. Our results showed that climate change would increase water supply by 4.85–8.16% and water demand by 1.45–2.36% in the B&R countries by 2050s. As a result, climate change will, in general, have little effect on water stress in the B&R countries as a whole, but it will exacerbate water shortage in some of the countries, especially in Central and West Asia. In addition, more countries are likely to suffer more severe water stress than countries benefiting from climate change. Different climate change scenarios had similar impacts on water stress in the B&R countries with a slightly greater number of countries suffering from more water stress under RCP 8.5 than RCP 4.5 and RCP 2.6.

## Supplemental Information

10.7717/peerj.12201/supp-1 Supplemental Information 1 Data sources.

10.7717/peerj.12201/supp-2 Supplemental Information 2 Country-based renewable water resources (1986–2005) and changes of renewable water resources under different climate change scenarios.

10.7717/peerj.12201/supp-3 Supplemental Information 3 The formula of the Nash coefficient.

## Additional Information and Declarations

### Data Availability

The following information was supplied regarding data availability: The raw data are available in the Supplementary Files.
