# Supplementary material for: Impacts of climate change on water resources in the major countries along the Belt and Road
Source: PeerJ. 2021 Oct 12;9:e12201. doi: 10.7717/peerj.12201 (PMC8519177; doi:10.7717/peerj.12201)
Supplement: Supplemental Information 3 [file peerj-09-12201-s003.docx]

Supplemental Information for

Impacts of climate change on water resources in the major countries along the Belt and Road

**Panpan Du^1,2,3^, Ming Xu^1,2^, Renqiang Li^1^**

^1^ Key Laboratory of Ecosystem Network Observation and Modeling, Institute of Geographic Sciences and Natural Resources, the Chinese Academy of Sciences, Beijing, China

^2^ University of Chinese Academy of Sciences, Beijing, China

^3^ Sino-Danish Centre for Education and Research (SDC), Beijing, China

Corresponding Author:

Ming Xu^1,2^

Key Laboratory of Ecosystem Network Observation and Modeling, Institute of Geographic Sciences and Natural Resources, the Chinese Academy of Sciences, 11A Datun Road, Beijing, 100101, China

Email address: [mingxu@igsnrr.ac.cn](mailto:mingxu@igsnrr.ac.cn)

Renqiang Li^1^

Key Laboratory of Ecosystem Network Observation and Modeling, Institute of Geographic Sciences and Natural Resources, the Chinese Academy of Sciences, 11A Datun Road, Beijing, 100101, China

Email address: [renqiangli@igsnrr.ac.cn](mailto:renqiangli@igsnrr.ac.cn)

The Nash coefficient, used to evaluate the performance of the model, was calculated by the following formula:

$$Nash=1- \frac{\sum_{i=1}^{n} {( y_{i,obs}-y_{i,sim})}^{2}}{\sum_{i=1}^{n} {( y_{i,obs}-\bar{y_{obs}})}^{2}}$$

Where $y_{i,obs}$ is the ensembled runoff data from GCM at coarse spatial resolution around 2° (*Table 1*), $\bar{y_{obs}}$ is the mean value of total coarse runoff data and $y_{i,sim}$ is the downscaled result by random forest model at the spatial resolution of 0.1°.
